# What are the optimum components in a care bundle aimed at reducing post-operative pulmonary complications in high-risk patients?

**DOI:** 10.1186/s13741-018-0084-9

**Published:** 2018-04-17

**Authors:** Sophie V. Griffiths, Daniel H. Conway, POPC-CB Investigators, Belda F. Javier, Manfred Blobner, Stijn Blot, Guillermo Bugedo, Luca Cabrini, Jaume Canet, Davide Chiumello, Milo Engoren, Lluis Gallart, G. Cosío Borja, Claude Guérin, Izabela Jachymiak, Ib Jammer, Cristiane Lamas, Arystarch Makowski, Luiz Marcelo Malbouisson, D. Matamis, Ramani Moonesinghe, John Moore, Monty Mythen, C. Paugam-Burtz, Rupert Pearse, Paolo Pelosi, Jan Poelaert, Huibert Ponssen, Bodil Steen Rasmussen, Michael Sander, Douglas Schuerer, Gerald W. Smetana, Charlotte Summers, Daniel Talmor, Karin Valkenet, Jelena Veličković, Jean-Louis Vincent, Sherry M. Wren, Michael Sander, Ib Jammer, Michael P. W. Grocott, Ben C. Creagh-Brown

**Affiliations:** 1 Faculty of Medicine, University of Southampton, Southampton, SO16 6YD UK; 2 Department of Anaesthesia and Critical Care, Central Manchester Foundation Trust, M13 9WL, Manchester, UK; 3 Department of Anaesthesiology, Intensive Care Medicine and Pain Therapy, University Hospital Gießen, Giessen, Germany; 4 Department of Anaesthesiology and Intensive Care, Haukeland University Hospital, Bergen, Norway; 5 Department of Clinical Medicine, University of Bergen, Bergen, Norway; 6 Critical Care Research Group, Southampton NIHR Biomedical Research Centre, Southampton University Hospitals NHS Trust/University of Southampton, Southampton, SO16 6YD UK; 7 Intensive Care Unit, Royal Surrey County Hospital, Guildford, GU2 7XX UK; 8 Surrey Perioperative Anaesthetic Critical care collaborative group (SPACeR), FHMS, University of Surrey, Guildford, GU2 7XH UK

**Keywords:** Post-operative pulmonary complications, Nosocomial pneumonia, Intra-operative ventilation, Inspiratory muscle training, Care bundle, Delphi consensus

## Abstract

**Background:**

Post-operative pulmonary complications (POPC) are common, predictable and associated with increased morbidity and mortality, independent of pre-operative risk. Interventions to reduce the incidence of POPC have been studied individually, but the use of a care bundle has not been widely investigated. The purpose of our work was to use Delphi consensus methodology and an independently chosen expert panel to formulate a care bundle for patients identified as being at high of POPC, as preparation towards an evaluation of its effectiveness at reducing POPC.

**Methods:**

We performed a survey of members of the ESICM POIC section to inform a Delphi consensus and to share their opinions on a care bundle to reduce POPC, the POPC-CB. We formed a team of 36 experts to participate in and complete an email-based Delphi consensus over three rounds, leading to the formulation of the POPC-CB.

**Results:**

The survey had 362 respondents and informed the design of the Delphi consensus. The Delphi consensus resulted in a proposed POPC-CB that incorporates components before surgery-supervised exercise programmes and inspiratory muscle training, during surgery, low tidal volume ventilation with individualised PEEP (positive end-expiratory pressure), use of routine monitoring to avoid hyperoxia and efforts made to limit neuromuscular blockade, and post-operatively, deep breathing exercises and elevation of the head of the bed.

**Conclusion:**

A care bundle has been suggested for evaluation in surgical patients at high risk of POPC. Evaluation of feasibility of both implementation and effectiveness is now indicated.

## Background

Post-operative pulmonary complications (POPC) are defined as ‘a pulmonary abnormality that produces identifiable disease or dysfunction that is clinically significant and adversely affects the clinical course’ (Restrepo and Braverman 2015). They are common, with a reported incidence ranging from 2–40%, and adverse outcomes include mortality, with studies reporting that 1 in 5 patients with POPC will die within 30 days of surgery, and the average hospital stay is lengthened by 8 days (Restrepo and Braverman 2015). A recent international prospective study (LAS VEGAS) of 9864 patients suggested that 28% of patients are at “high risk” of developing POPC and in this cohort, the incidence of POPC is of 19.2%, with respiratory failure affecting 3.4% (Rogerson et al. 2017). Unplanned supplemental use of oxygen is the commonest POPC, and in another contemporary study, even this ‘mild’ POPC was associated with increased risk of mortality and morbidity (Fernandez-Bustamante et al. 2017).

Alongside other perioperative complications, POPC affect long-term outcomes, with patients having lower long-term survival rates following POPC independent of preoperative risk (Khuri et al. 2005). Furthermore, the economic burden of POPC is substantial, with a 55% increase in hospital costs in non-cardiac patients (Shander et al. 2011). Reduction in the rate of POPC starts with the recognition of moderate- and high-risk patients through the use of validated tools such as ARISCAT (Assess Respiratory rIsk in Surgical patients in CATalonia) (Canet et al. 2010; Canet et al. 2015).

Enhanced recovery (ER) pathways are advocated as ‘standard of care’ for major surgery, and there are pathways specific to each surgical specialty (Ljungqvist et al. 2017). Several aspects of the pathways are either established to be associated with reduced POPC (minimally invasive surgery (Lee et al. 2015)) or strongly suspected of being associated with reduced POPC (early mobilisation (Pasquina and Walder 2003)). Adherence to ER components is highly variable, and improved adherence has been associated with improved outcomes (Pecorelli et al. 2017).

Several perioperative interventions aiming to reduce the incidence of POPC have been studied sometimes in combination. A care bundle (CB) is a group of interventions that when used together have greater clinical effect than when used individually. A CB for POPC used in the ‘I COUGH’ study demonstrated a trend towards reduction in POPC, and CBs have significantly improved outcomes in the critical care environment to prevent ventilator-associated pneumonia (Rello et al. 2013).

The Delphi consensus method was first introduced by the RAND corporation in Santa Monica in the 1950s. The method involves gathering expert opinions through rounds of questions and bringing these opinions together to establish a consensus. Each round is followed by anonymous feedback to all participants, and the results of the previous round are then used to create the subsequent round. This means that over the rounds, a consensus can be derived which is as reliable as possible (Dalkey and Helmer 1963).

Our primary objective was to recruit a team of experts to participate in an electronic Delphi consensus, leading to the formulation of a perioperative care bundle ‘POPC-CB’ intended to reduce the incidence of POPC. The POPC-CB was designed to be applied in addition to, rather than replacing, enhanced recovery pathways in patients at moderate to high risk of POPC.

## Methods

We performed a survey of members of the European Society of Intensive Care Medicine (ESICM) Perioperative Intensive Care (POIC) section, both to identify ‘expert volunteers’ for the Delphi and to gain an appreciation of clinical opinion regarding the potential components of a POPC-CB. From a survey sent to the 6623 members of the POIC group of ESICM, there were 362 responses which helped establish the potential of POPC risk assessments and possible interventions in a POPC-CB for patients screened at moderate to high risk. One hundred thirty-four experts were identified during this process, and they were invited to collaborate with the project. Additionally, 41 authors from key literature on the subject were invited to participate and 38 experts agreed to form the consensus group.

Three rounds of questions were anonymously circulated to the Delphi group via email with a 2-week deadline for responses to each round. The questions comprised a series of statements which the experts were asked to respond to using a 5-point Likert scale alongside free text spaces for comments and references to relevant publications. Some rounds included more open-ended questions and a ranking system for a preferred option. If an expert wanted to skip a question, the response box would be left blank. After each round, the project coordinators collated the results of the round and feedback of themes was given to the group. By the end of the process, the final feedback was sent to the group, this time not anonymised, with the consensus and a suggested care bundle.

## Results

### Survey

Three hundred sixty-two members of the POIC group participated in the initial survey. Routine screening for POPC risk occurred in 50% of centres, with varying POPC reduction techniques available (Appendices 1 and 2). Seventy-eight percent of respondents specified that they would be happy to participate in developing and assessing the implementation of a POPC-CB.

### Delphi

Three rounds of the Delphi were circulated; in total, 35 experts responded to at least one round with 21 replying to all rounds. Two experts who expressed interest in being involved in the project did not reply to any of the rounds, and one expert withdrew from the consensus.

POPC encompasses a range of clinical syndromes (i.e. respiratory failure) and diagnoses (i.e. pneumothorax), and there are limitations in having this as an endpoint. However, there was a strong consensus (> 90% strongly agreeing (SA) or agreeing (A)) that reduction in POPC as a composite was the goal of the CB. There was a strong consensus (28/29, 97%) that the POPC-CB should be applied to higher-risk patients (as opposed to all patients) and include pre-, intra- and post-operative components *in addition* to standard enhanced recovery elements (100%). There was no consensus on the minimum required specific interventions that any patient should receive in order for them to have consider to have been treated within an ERAS pathway. ERAS implementation varies between centres and between types of surgery. It was acknowledged that various methods can be used to identify patients at higher risk of POPC and the ARISCAT score was the most widely recognised validated screening tool. Less popular were the Arozullah (Arozullah et al. 2000) and Gupta methods (Gupta et al. 2011). It was agreed to design the POPC-CB without cost being a major consideration.

Following a round whereby we gained information about what types of intervention were favoured during each perioperative stage, we progressed to better refining which components were the most favoured (Table 1). There was a weak consensus that seven was the optimal number of components in the POPC-CB.

**Table 1:** Table to demonstrate the components included in the CB and those which were not chosen by the Delphi consensus

	Pre-operative	Intra-operative	Post-operative
In the care bundle	• Supervised exercise programme • Inspiratory muscle training (^*^)	• Low tidal volume ventilation (^**^) with individualised PEEP • Use of routine monitoring to avoid hyperoxia • Limit NM blockade	• Deep breathing exercises • Mandatory elevation of the head of the bed
Not in the care bundle	• Oropharyngeal decontamination • Oral care package • Chlorhexidine mouthwash or other selective oral decontamination • Selective digestive decontamination • Incentive spirometry • Deep breathing exercises • Daily pedometer targets	• Recruitment manoeuvres • Routine use of high levels of PEEP • Use of endotracheal tubes with specific design features, including subglottic secretion drainage • Specific drugs or techniques to limit residual neuromuscular blockade	• Prophylactic ventilator support including CPAP, NIV or high-flow nasal oxygen • Pharmacological therapies that aim to decrease gastro-oesophageal reflux

A recommendation with (^*^) moderate quality or (^**^) strong quality evidence using GRADE or Jadad criteria

## Discussion

We used a Delphi process to design a care bundle (POPC-CB), for patients at moderate to high risk of post-operative pulmonary complications which may have the potential to improve clinical outcomes. The proposed interventions are implemented throughout the perioperative journey: prior to the operation, supervised exercise programme and inspiratory muscle training, and during the operation, low tidal volume ventilation with individualised PEEP, use of routine monitoring to avoid hyperoxia and efforts made to limit neuromuscular blockade, and also post-operatively, deep breathing exercises and mandatory elevation of the head of the bed.

The quality and extent of clinical evidence to support each of the proposed components of the POPC-CB is variable. The Delphi process did not involve a formal evaluation of the strength of evidence for the interventions included in the care bundle, nor for those that were rejected. Some of the individual interventions have been evaluated by others using GRADE or Jadad methodologies (Guyatt et al. 2011; Jadad et al. 1996). A Cochrane review of preoperative inspiratory muscle training demonstrated efficacy at reducing POPC (Katsura et al. 2015). Using GRADE criteria, the Cochrane reviewers suggested the strength of evidence for pneumonia reduction was moderate whilst the strength of evidence for atelectasis and adverse event reduction was low and recommended caution due to the potential for overestimation of treatment effect. Although our Delphi process supports the inclusion of supervised preoperative exercise programme, the evidence for improved postoperative clinical outcomes is limited (O’Doherty et al. 2013). A meta-analysis of nine prehabilitation trials in 435 abdominal surgery patients suggests that supervised exercise can reduce overall postoperative complications, and five of these studies specifically demonstrated a reduction in postoperative pulmonary complications (Moran et al. 2016). A very low quality rating was observed using GRADE criteria due to the small sample sizes and significant risk of bias. Furthermore, the precise components of such a supervised programme have not been fully evaluated in relation to POPC reduction.

Protective lung ventilation strategies incorporating tidal volumes 5–8 mL/kg, higher PEEP and control of plateau pressure are beneficial in critically ill patients with acute lung injury or ARDS (Villar et al. 2006; Petrucci and Iacovelli 2007). In ICU patients without ARDS, a protective lung strategy reduced pulmonary and systemic inflammation and reduced the development of lung injury (Determann et al. 2010). A meta-analysis of 20 studies, using GRADE criteria, suggested the evidential strength for protective lung strategy in patients without ARDS to be moderate and high in reducing lung injury and mortality, respectively, but low in preventing pneumonia (Neto et al. 2012). These findings from critical care patients led to perioperative research exploring whether protective lung ventilation has a role during major surgery. The IMPROVE trial compared a ‘protective’ strategy of tidal volume 6–8 mL/kg ideal body weight, PEEP 6–8 cm H_2_O, recruitment manoeuvers every 30 min with ‘conventional’ management of tidal volume 10–12 mL/kg, and PEEP and recruitment manoeuvers at clinicians’ discretion. The protective strategy was associated with a reduction in a composite of pulmonary and septic complications (Futier et al. 2013a). In another trial, PROVHILO, all patients received tidal volumes 8 mL/kg and were randomly assigned to either rather high PEEP of 12 and regular recruitment manoeuvers or to low PEEP 0–2. The result shows no difference in post-operative pulmonary complications but shows increased adverse events in the high PEEP group (Prove Network Investigators for the Clinical Trial Network of the European Society of Anaesthesiology et al. 2014). Even in critically ill ventilated ARDS patients, high versus very high PEEP levels was not shown to influence outcome (Brower et al. 2004). A meta-analysis of 15 studies investigating protective lung ventilation involving 2127 surgical patients concluded that intraoperative low tidal volume is associated with less pulmonary complications but no difference in mortality and length of hospital stay (Neto et al. 2015). The authors used the Jadad criteria to rate the quality of this evidence with seven studies scoring 4/5, seven 3/5 and one 2/5. Our Delphi consensus suggests low tidal volumes for all patients with individualised PEEP and recruitment manoeuvres at the discretion of the attending anaesthetist.

Inadequate reversal of neuromuscular blockade is associated with POPC. Post-operative residual curarization (PORC) is the incomplete recovery of muscle function following perioperative use of neuromuscular blocking agents. The prevalence of PORC is anything between 4 and 45%, when defined as train-of-four < 0.9 and is associated with adverse outcomes including POPC (Berg et al. 1997). Whilst the Delphi consensus recommendation is to limit neuromuscular blockade by careful monitoring of neuromuscular function and effective reversal, we acknowledge that ongoing research assessing the impact of neuromuscular blockade on POPC is awaiting publication and no specific drug or technique can be recommended. The potential for hyperoxia to cause harm is contentious. The association between hyperoxia and atelectasis is well established, although the magnitude of this effect may be overestimated (Pryor et al. 2004). A meta-analysis of 17 randomised controlled trials with 8093 mixed surgical patients used the Jadad score to assess quality and found six studies scored 5/5, four 4/5 and 7 less than 4, with sub-group analysis suggesting that hyperoxia can reduce surgical site infection in colorectal surgery patients (Yang et al. 2016).

Post-operative care from a specialist physiotherapist is a commonplace; however, the interventions delivered by physiotherapists vary significantly, and there is a scarcity of good-quality clinical evidence to inform practice with many small, underpowered studies using inadequate randomisation and unclear interventions (ÖRman and Westerdahl 2010; Pasquina et al. 2006). The incremental benefit of the proposed deep breathing exercises is uncertain, particularly when other aspects of post-operative physiotherapy, such as early mobilisation, are provided (Mackay et al. 2005). A Cochrane review of post-operative incentive spirometry following upper abdominal surgery described a small number of poor-quality studies which were unable to demonstrate any effect on postoperative complications (do Nascimento Junior et al. 2014). Elevation of the head of the bed, allowing gravity to help minimise pulmonary aspiration of oropharyngeal secretions and perhaps improve effectiveness of coughing, has been shown to reduce ventilator-associated pneumonia in critically ill patients although no evidence is available in POPC reduction (Wang et al. 2016).

I COUGH, a post-operative pulmonary care bundle, demonstrated a trend towards reduction in POPC (Cassidy et al. 2013). I COUGH incorporates Incentive spirometry, Coughing and deep breathing, Oral hygiene, Understanding (patient education), Get up (mobilisation) and Head of bed elevation. I COUGH was applied to all patients, not just those at high risk of POPC, and it was evaluated in a single centre and was evaluated prior to widespread adoption of enhanced recovery pathways. Our POPC-CB is designed to supplement established guidelines for enhanced recovery, not replace them (Lassen et al. 2009; ERAS society guidelines n.d.). Although this protocol is designed for all patients undergoing major surgery, elements of ERAS pathways are relevant to reducing the incidence of POPC. These include optimal analgesia, avoidance of nasogastric tubes and bowel preparation, minimisation of ‘nil by mouth’, avoidance of sodium and water overload, routine early post-operative mobilisation, multi-modal analgesia and prevention of nausea and vomiting. A quality improvement programme incorporating ICOUGH into ERAS pathways has reduced POPC over time (Moore et al. 2017).

### Strengths and limitation**s**

An expert consensus using Delphi techniques is fundamentally dissimilar to a systematic review of evidence, and this is both a strength and a weakness. We made no attempt to inform or influence the experts, and the POPC-CB reflects opinions not necessarily supported by clinical evidence. Several components in this proposed CB have been the subject of detailed studies whilst others such as high-flow nasal oxygen and continuous positive airway pressure are under evaluation (Futier et al. 2013b). Conversely, some interventions that were considered but not selected for the POPC-CB have good evidence of clinical efficacy such as perioperative oral decontamination (Spreadborough et al. 2016).

During the Delphi process, ‘avoiding hyperoxia’ (excessively high partial pressure of oxygen, measured directly or detected from pulse oximetry) as an intra-operative component proved controversial leading to one expert withdrawing from the consensus amid concerns that some clinicians may misinterpret this component as ‘permissive hypoxaemia’ or avoidance of high inspired oxygen during critical airway interventions such as intubation. We believe that avoiding hypoxaemia is a fundamental part of anaesthetic management and therefore should not require inclusion within the POPC-CB.

### Research recommendations

Prior to an evaluation of the clinical effectiveness of this POPC-CB, it is necessary to gain more information about the uptake of enhanced recovery programmes and, more specifically, the feasibility of undertaking this study. Only 50% of survey respondents screen patients for POPC risk, and a consistent screening of all patients needs to be implemented. Consistent delivery of all aspects of the care bundle will need to be specified, for example, what is meant by ‘supervised exercise programme’. It is uncertain to what extent these measures are part of routine clinical practice. Recent data from the UK suggest that protective lung ventilation in major open abdominal surgery is rarely used (Patel et al. 2016). Robust collection of post-operative outcome data, using standardised measures, is essential, and this may require additional local resources (Myles et al. 2016). Alternatively, large databases of surgical patients could potentially relate provision of POPC-CB components to the incidence of POPC.

## Conclusion

Patients at moderate to high risk of POPC, undergoing major elective surgery within an enhanced recovery programme, can be identified using screening tools such as the ARISCAT score and may benefit from a care bundle aimed at reducing these complications such as that described during our Delphi process. We believe a multi-centre international evaluation of the efficacy of such a care bundle in reducing the incidence of POPC, length of stay and long-term outcomes should be a priority for research in perioperative care.

## Figures and Tables

**Fig. 1:**
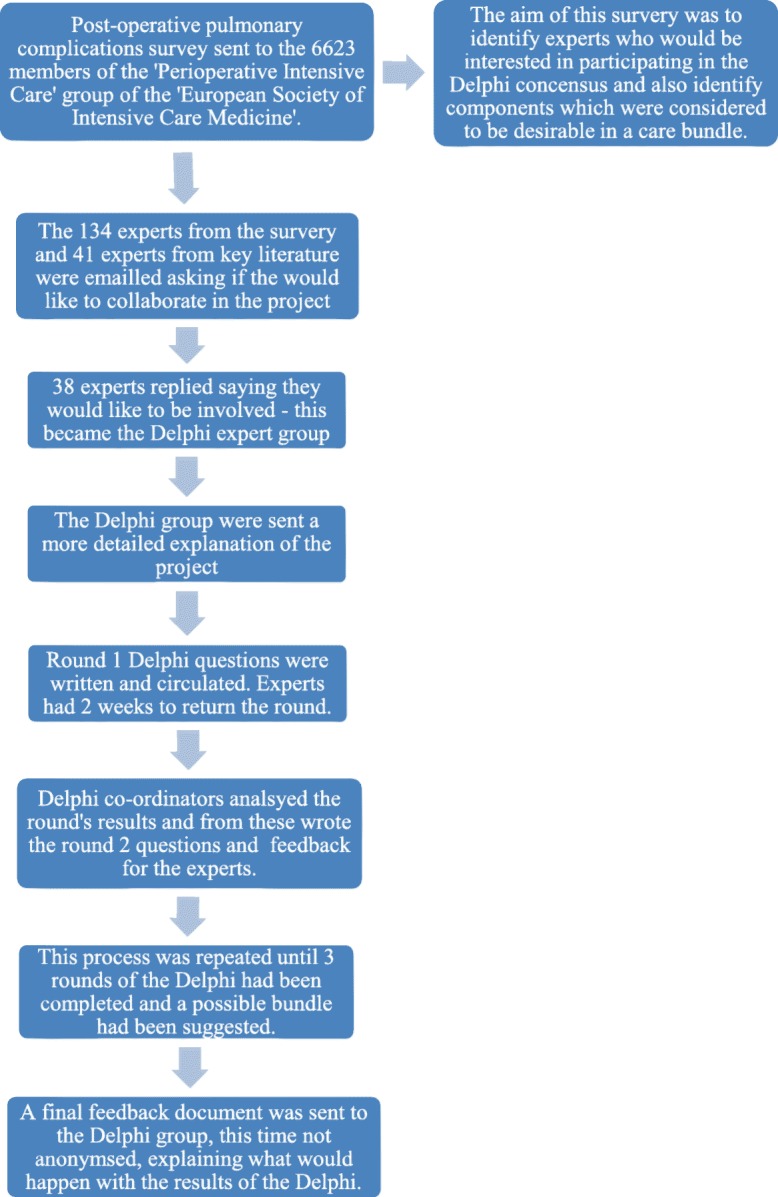
Flow diagram to summarise the methods

## Appendix 2

### Results of the POPC survey of the POIC group of the ESICM

POIC group (http://www.esicm.org/sections/perioperative-intensive-care) is a special interest section for members of the European Society of Intensive Care Medicine (ESICM). There are 6623 members and a link to a survey monkey was emailed to them during October 2015. There were 362 responses (5.5%).

**Table:**

Question 1: In your institution, is it routine to screen patients undergoing surgery to identify those at high risk for POPC?		
Answer options	Response count	Response percentage (%)
Yes	174	48.7
No	183	51.3

Question 2: If institutions were to routinely screen for patients at high risk for POPC and then provide a POPC reduction care bundle, what recommendations would you make for constituents of this care bundle? To be applied *PRE-operatively*:		
Answer options	Response count	Response percentage (%)
Chlorhexidine mouthwash	155	50.5
Selective oral decontamination (SOD), e.g. anti-microbial paste	34	11.1
Selective digestive decontamination (SDD), e.g. non-absorbable enteral antibiotics and IV antibiotics	24	7.8
Oral care, e.g. toothbrushing, dental hygienist review etc.	147	47.9
Supervised exercise programme, also known as pre-habilitation	140	45.6
Nebulised/inhaled medical therapy	94	30.6
Enteral/intravenous medical therapy	39	12.7
Incentive spirometry or positive end-expiratory pressure valve	197	64.2
Inspiratory muscle training	169	55.0

Question 3: If institutions were to routinely screen for patients at high-risk for POPC and then provide a POPC-reduction care bundle, what recommendations would you make for constituents of this care bundle? To be applied *INTRA-operatively*:		
Answer options	Response count	Response percentage (%)
Low tidal volume ventilation, e.g. 6–8 mL/kg tidal volumes	286	84.6
Appropriate PEEP	251	74.3
Regular recruitment manoeuvres	141	41.7
Endotracheal tube with subglottic secretion drainage	101	29.9
Nebulised antibiotics	9	2.7
Avoid blood transfusion by tolerating anaemia unless Hb ≤ 70 g/L	193	57.1
Use humidified ventilator circuit	182	53.8
Avoid neuromuscular blockade (NMB) other than short-acting NMB for intubation	147	43.5
Specific methods to evaluate or reverse NMB	117	34.6
Fluid resuscitation using advanced monitoring (EVLWI or lung US) to avoid pulmonary oedema	147	43.5
Avoid hyperoxia (saturations ≥ 99% or PaO_2_ ≥ 14 kPa)	206	60.9
Anti-sialogogues, e.g. glycopyrronium	40	11.8

Question 4: If institutions were to routinely screen for patients at high risk for POPC and then provide a POPC-reduction care bundle, what recommendations would you make for constituents of this care bundle? To be applied *POST-operatively*:		
Answer options	Response count	Response percentage (%)
High-flow humidified oxygen via nasal cannulae	158	46.3
CPAP administered via facemask or hood	174	51.0
Extubation directly onto bi-level positive pressure ventilation via face mask in all patients	38	11.1
Extubation directly onto bi-level positive pressure ventilation via face mask in selected patients	105	30.8
Incentive spirometry/regular use of positive end-expiratory pressure valve	187	54.8
Inspiratory muscle training	162	47.5
Specific physical therapy or exercises, additional to ERAS early mobilisation	147	43.1
Avoid use of gastric acid suppression medications	96	28.2
Head of bed elevation	309	90.6
Mini-tracheostomy to permit suctioning of airway secretions	13	3.8
Avoidance of hyperoxia (saturations ≥ 99% or PaO_2_ ≥ 14 kPa)	194	56.9

Question 5: Would you potentially be interested in assessing the feasibility of using this care bundle approach in patients who are at high risk of POPC in your institution?		
Answer options	Response count	Response percentage (%)
Yes	242	79.1
No	64	20.9

Question 6: Once feasibility has been assessed, might your institution be interested in participating in an international multi-centre clinical trial assessing the effectiveness of a POPC reduction care bundle?		
Answer options	Response count	Response percentage (%)
Yes	239	78.1
No	67	21.9
